# Behavioral Predictors of Adolescent Anxiety During Therapy Dog Interactions Within an Experimental Setting

**DOI:** 10.3390/bs16030391

**Published:** 2026-03-09

**Authors:** Nicole Mason, Seana Dowling-Guyer, Eric C. Anderson, Megan K. Mueller

**Affiliations:** 1 Center for Animals and Public Policy, Cummings School of Veterinary Medicine at Tufts University, Grafton, MA 01536, USA; seana.dowling_guyer@tufts.edu (S.D.-G.); megan.mueller@tufts.edu (M.K.M.); 2 Center for Interdisciplinary Population and Health Research, MaineHealth Research Institute, Portland, ME 04074, USA; 3 Tufts University School of Medicine, Boston, MA 02111, USA

**Keywords:** animal-assisted interventions, dog behavior, human behavior, affiliative, stress, anxiety

## Abstract

Animal-assisted interventions (AAIs) are emerging as a promising treatment avenue for adolescent social anxiety. However, the behavioral mechanisms underlying positive results remain unclear. In addition, behavioral signs of discomfort or stress in therapy dogs may suggest concerns about the dog’s welfare. This study examined the associations between stress-linked and affiliative behaviors of adolescents and therapy dogs with adolescent stress reactivity outcomes within an experimental setting in a sample of 50 participants. Linear regression models primarily indicated null findings, with no significant relationships between adolescent affiliative, dog affiliative, or adolescent stress behaviors to self-reported anxiety or psychophysiological arousal. However, the stress-linked behaviors of shake-off and yawning in dogs were negatively associated with adolescent stress reactivity, although the relationships had small effect sizes. These findings provide preliminary insights into the behavioral mechanisms related to changes in adolescent arousal and implications for dog welfare within AAIs. Future research should replicate with larger samples, test for effects with different dogs, and use diverse physiological stress markers for both species involved in the intervention. Additionally, examining the temporal relationships between adolescents and therapy dog behaviors to stress reactivity outcomes would provide insight into causal relationships.

## Simple Summary

Animal-assisted interventions are growing in popularity as a potential treatment option for adolescents with social anxiety. However, research on the behaviors between adolescents and therapy dogs that lead to changes in adolescent anxiety is limited. This study aimed to examine how stress-linked and affiliative behaviors in both adolescents and dogs might be associated with participant stress reactivity within an experimental setting. The primary results indicated that most adolescent–dog behaviors measured in this study were not associated with adolescent reactivity. However, dog shaking-off and yawning behaviors were negatively associated with changes in adolescent arousal, though the statistical relationships were weak. While replication and expansion are needed to draw generalizable conclusions, this study provides initial insight into the role of adolescent–dog interactions within animal-assisted interventions.

## 1. Introduction

Social anxiety is a common mental health concern for adolescents that affects 8.3% of the global population (Salari et al., 2024). Marked by the extreme fear of scrutiny within social situations, social anxiety can result in behaviors such as avoidance of interactions with peers, which can be detrimental to adolescent positive development (American Psychiatric Association, 2013; Chiu et al., 2021). Given the impact that social anxiety can have on an adolescent’s ability to develop the social skills needed for positive adult functioning, there is a pressing need to explore how targeted interventions can reduce anxiety.

Animal-assisted interventions (AAIs) are growing as a promising strategy to support adolescent coping with social anxiety (Brandão et al., 2025). Previous research has shown that including dogs in therapeutic interactions can provide social support to youth by promoting prosocial engagement and building interpersonal communication skills (Flynn et al., 2025), buffering social stress (O’Haire et al., 2015), and regulating physiological stress reactivity (Gandenberger et al., 2024). However, the specific behaviors that may support beneficial outcomes in AAIs for socially anxious youth remain underexplored (Dowling-Guyer et al., 2024).

A key component of understanding the adolescent–dog dyadic behaviors within AAIs is exploring the role that stress-linked and affiliative behaviors play within the intervention (Dowling-Guyer et al., 2024). However, further research is needed to examine how these behavioral patterns emerge within therapy dog interactions across human populations and intervention types, and if the therapy dog behavior may impact changes in anxiety for adolescents with social anxiety. Research on therapy dogs’ experiences of stress or discomfort while engaging in AAIs remains inconclusive (Erichsmeier et al., 2025; Glenk, 2017), which could be a point of concern when considering their welfare during the therapeutic interaction (Dowling-Guyer et al., 2024). Thus, this study seeks to fill the gap by examining stress-linked and affiliative behaviors between adolescents with social anxiety and therapy dogs within an experimental AAI setting.

### 1.1. Adolescent Social Stress and Animal-Assisted Interventions

As quality peer relationships are a key milestone for positive adolescent development, identifying interventions to support adolescents’ ability to manage interpersonal stressors is crucial. An avenue that is gaining momentum as a potential intervention to support adolescents’ ability to cope with social stress is AAIs (Brandão et al., 2025; Kertes et al., 2017). AAI is a commonly used umbrella term for a broad range of therapeutic interactions or interventions involving animals (Etheridge, 2019; International Association of Human-Animal Interaction Organizations, 2018). Additional phrases used to describe these interactions are also called “animal-assisted services,” “canine-assisted interventions” (Green et al., 2024), or “therapeutic interactions that include dogs” if using service-first language guidelines (Wood et al., 2021). While recognizing the evolving guidance on terminology use, this manuscript will refer to the structured therapeutic interaction between a person and a dog as AAI in order to best align with existing research on this topic (Green et al., 2024).

Prior research suggests that AAIs can assist with a range of youth mental health conditions (Hoagwood et al., 2017), including their ability to cope with stress and anxiety (Sim et al., 2025). These beneficial outcomes of AAIs are of particular interest in treating adolescent social anxiety, as this population has a low rate of pursuing professional help for their symptoms (Burstein et al., 2011). Additionally, incorporating dogs into therapeutic settings has been linked to increased youth motivation to engage in mental health treatment (Flynn et al., 2022).

### 1.2. Mechanistic Drivers of Effective Treatment in AAI

While incorporating dogs into therapeutic settings has shown positive outcomes in addressing adolescent mental health (Hoagwood et al., 2017), the mechanisms driving those benefits are still unclear. One theory of the underlying drivers behind the positive outcomes within AAIs is that the animal provides a social resource (Flynn et al., 2025; Kretzler et al., 2022; Martins et al., 2023; Sim et al., 2025). In a recent qualitative meta-synthesis, Flynn et al. (2025) reported that including dogs in therapeutic interactions for youth encouraged prosocial engagement and the development of interpersonal communication skills among peers. Several studies have also highlighted the role dogs can play in buffering physiological responses to social stress (Gandenberger et al., 2024; Kertes et al., 2017; O’Haire et al., 2015). Indeed, recent research found a significant reduction in momentary stress among college youth in a social situation when they were able to touch a therapy dog (Binfet et al., 2021). However, findings remain inconclusive regarding how behavioral interactions with dogs relate to reductions in anxiety amongst adolescents experiencing social stress (Mueller et al., 2021).

To determine whether a therapeutic approach is effective in treating a mental health condition within a specific population, one important step is to identify the interactive mechanisms within the intervention that are associated with positive outcomes (Dowling-Guyer et al., 2024; Nieforth et al., 2024). Yet, the identification of human and therapy animal behavioral dynamics is an underexplored area in AAI research (Dowling-Guyer et al., 2024). A few recent studies have attempted to fill this gap by exploring the behavioral dynamics between children and therapy dogs (Kilmer et al., 2025; Lee et al., 2022; Nieforth et al., 2024). However, these aforementioned studies did not examine both human and dog behaviors within an AAI that is targeting a specific mental health condition. As the therapy dogs play a fundamental role in AAIs, understanding their part in treatment effectiveness requires examining their behavioral responses to the intervention.

Using data from an experimental AAI study addressing social stress in adolescents (Mueller et al., 2021), Dowling-Guyer et al. (2024) recently examined the behavior of both adolescents with social anxiety and therapy dogs during a laboratory stress task. The authors noted that an area for future research is to examine these adolescent–dog interactions within the context of stress-linked (e.g., yawning, panting) or affiliative (e.g., wagging tail, sniffing a participant) behaviors, as these interpersonal responses may be related to treatment outcomes. Affiliative behaviors are typically associated with social bonding across species (Stoesz et al., 2013). For example, affiliation has been observed between best friendships amongst adolescents (Martin et al., 2017). Affiliative behaviors have also been linked to positive social interactions between dogs (Duranton, 2020).

Given the impact affiliative behaviors have on bonding across human and dog species interactions, it is important to understand the role these types of behaviors play within AAIs and the resulting impact on treatment efficacy. For example, in relation to social anxiety, an affiliative response from a therapy dog could be an avenue through which the client receives social and emotional support. Adolescent affiliative behaviors may also relate to bond development with dogs during the therapeutic interactions, as affiliation has been linked to human–dog relationship development (Payne et al., 2015). Alternatively, examining the interactive dynamics of adolescent stress behaviors within AAIs can provide insight into factors that may be impacting unfavorable treatment outcomes (Dowling-Guyer et al., 2024). While most studies in AAI that target adolescent social stress relate to overall efficacy of the intervention (Brandão et al., 2025), little research examines what factors may drive positive, null, or negative effects (Dowling-Guyer et al., 2024).

Stress-linked behaviors exhibited by therapy dogs could be considered both a component of treatment efficacy and a welfare concern. There is a growing push within the HAI field to incorporate a OneWelfare framework into future research, which asserts the understanding that the well-being of humans, animals, and the environment is intrinsically intertwined (Hoy-Gerlach & Townsend, 2023; International Association of Human-Animal Interaction Organizations, 2018). Given that the animal is a core component of AAI, it is imperative to consider the well-being of both dog and human participants with equal regard (International Association of Human-Animal Interaction Organizations, 2018). Consideration for both human and dog outcomes is crucial, not just for gaining insight into treatment effectiveness, but also for ensuring the physical and emotional well-being across the species involved within the intervention. Furthermore, by prioritizing well-being across both human and animal domains within AAI, the field can work towards ensuring mutually beneficial research and practice standards (Leconstant & Spitz, 2022; Santaniello et al., 2021).

As concern for dog well-being within AAI has gained traction, several studies have examined the effects of dogs’ stress while involved in therapeutic interactions (Gandenberger et al., 2022; Sidel et al., 2025) including stress-linked (Lee et al., 2022), and displacement behaviors (Erichsmeier et al., 2025). However, what is missing is an understanding of how those stress-linked behaviors relate to participant outcomes. Should a dog demonstrate stress-linked behavior during the intervention, like shifting away from a participant during an interaction, this could potentially have a negative impact on treatment effectiveness (Dowling-Guyer et al., 2024). This moving-away behavior from the dog could also be interpreted by a socially anxious adolescent as the dog perceiving their interaction negatively. This could be a plausible assumption, as a hallmark of social anxiety is experiencing extreme self-criticism within social contexts (Haller et al., 2015). Thus, research is needed to examine both the affiliative and stress-linked behaviors between socially anxious adolescents and therapy dogs within an AAI in order to investigate how those dyadic behaviors relate to treatment outcomes and dog welfare.

### 1.3. The Present Study

There is growing evidence to suggest that AAIs may be a viable treatment option to address adolescent social anxiety (Brandão et al., 2025; Kertes et al., 2017), but the behavioral mechanisms driving potential effects on anxiety have not yet been fully explored (Mueller et al., 2021). Additionally, while the dogs are a critical component of the intervention, the experience of the therapy dog can often be overlooked in research on AAIs (Glenk, 2017; Glenk & Foltin, 2021; Sidel et al., 2025). Thus, we examined adolescent–dog interactions through the framework of stress-linked and affiliative behaviors to determine how these interactions relate to AAI treatment effectiveness, as measured by adolescent self-reported anxiety and physiological arousal prompted by an experimental stressor. To achieve this, we used data from Mueller et al.’s (2021) randomized control trial (RCT) that tested the effects of a social stress task on adolescents with social anxiety within an AAI. We aimed to investigate the predictive relationship between adolescent–dog affiliative and stress-linked behaviors and adolescent outcomes within the experimental AAI. Ultimately, we tested whether there were signs of a predictive relationship between the variables, not to examine whether causality between the behaviors and changes in adolescent stress reactivity outcomes existed.

We had two primary hypotheses being tested in this study:

**Hypothesis** **1:** *Adolescent and dog stress-linked behaviors would be positively associated with adolescent peak anxiety and negatively associated with anxiety recovery. We anticipated that, as both adolescents and therapy dogs displayed stress-linked behaviors, we would see a greater peak in adolescent anxiety and a lower magnitude of recovery.*

**Hypothesis** **2:** *Adolescent and dog affiliative behaviors would be negatively associated with adolescent peak anxiety and positively associated with anxiety recovery. As we observed greater incidences of affiliative behaviors between adolescent–dog interactions, we expected lower levels of adolescent anxiety and greater recovery after the stressor.*

## 2. Methods

**Figure 1:**
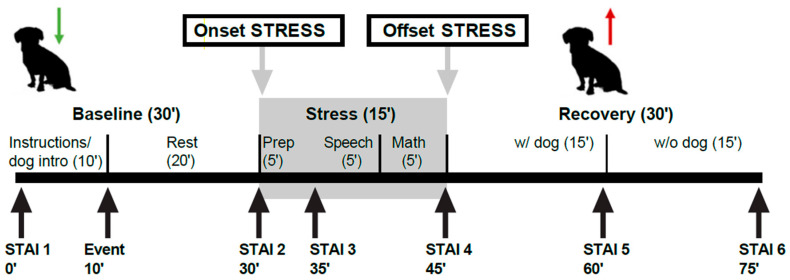
Original study timeline and tasks as published in Dowling-Guyer et al. (2024). Notes: STAI = State Trait Anxiety Inventory. Reproduced with modifications with permission from Seana Dowling-Guyer, Behavior Coding of Adolescent and Therapy Dog Interactions During a Social Stress Task.

This present study used data from the experimental conditions within Mueller et al.’s (2021) clinical trial of an AAI for adolescents with social anxiety who have undergone a social stress task (see Figure 1). Only the data from the experimental groups were used as they were the only participants exposed to a live therapy dog. The data collected for this present study included adolescent electrodermal activity (EDA) and self-reported momentary anxiety that was captured through a brief survey measure, as well as video behavioral data for both the adolescents and the therapy dogs involved in the trial. The coding of this behavioral data was conducted and reported by Dowling-Guyer et al. (2024). The procedures of the original trial (Mueller et al., 2021) are outlined below.

The original procedures were approved by the Tufts University Institutional Review Board (protocol #1702004), the Institutional Animal Care and Use Committee (protocol #G2017-09), and the study was registered on ClinicalTrials.gov (ID: NCT03249116). Procedures for the video coding of the adolescent and dog behaviors conducted by Dowling-Guyer et al. (2024) were approved by the Tufts University Review Board (protocol #1599).

### 2.1. Participants

The present study only uses data collected from the 50 adolescents (ages 13–17) from Mueller et al.’s (2021) experimental (live therapy dog) conditions and does not include the no dog control condition, due to this study’s focus on dog behavior. Adolescents had a mean age of 15.52 (*SD* = 1.46)*,* were primarily female (80%, *n* = 40), White (88%, *n* = 44), and not of Hispanic or Latin ethnic descent (92%, *n* = 46). Adolescents were eligible to participate if they were 13–17 years old, were not allergic or fearful of dogs, and did not have any allergies related to adhesives in order to wear the electrodermal activity sensor. Participants were screened for social anxiety via the Social Anxiety Scale for Adolescents (SAS-A) (La Greca & Lopez, 1998).

The therapy dogs and their handlers were recruited from Tufts Paws for People, which registers all therapy dog and their handlers through Pet Partners^®^, the international accrediting body for volunteer therapy dog teams (Pet Partners, n.d.). Each registered therapy dog team (dog + their handler) must go through extensive training and pass a proficiency exam in order to become certified. A total of four certified therapy dog teams were recruited for this trial. The characteristics of the therapy dogs included three female and one male dog, all of which weighed 30 pounds or less, were middle to older in age (age 8–13), and were spayed/neutered. Therapy dogs were four different breeds or breed mixes: shi-tzu, terrier mix, miniature poodle, and a Labrador beagle mix. Dog owners provided informed consent for their dogs’ participation in the study.

### 2.2. Procedures

After completing the SAS-A screener (Mueller et al., 2021), participants were categorized as having low (*n* = 10), mid-range (*n* = 15), or high levels of social anxiety (*n* = 25) based on guidelines from La Greca and Lopez (1998). Participants within each level of social anxiety were then randomized into either of the two experimental conditions, including: (1) social and physical interaction with a live therapy dog (*n* = 25) or (2) social interaction but with no physical contact with the live therapy dog (*n* = 25) as part of the original study hypotheses (along with an additional 25 participants in a no-dog control group not included in these analyses). This was done to ensure an even distribution of social anxiety levels across all conditions.

Each participant completed the Trier Social Stress Task for Children (TSST-C), which was the experimental stimulus used to provoke adolescent social stress. The TSST-C is a widely used experimental paradigm for assessing social stress and comprises six phases, including: baseline, anticipation, preparation, speech, mental math and recovery (Buske-Kirschbaum et al., 1997; Stroud et al., 2009). The baseline, anticipation, and preparation phases are used to prime the adolescent for the stress task. The experimental phases asked the participants to engage in public speaking and answer mathematical questions to induce stress. The last phase of the TSST-C involved a 30 min recovery period. This recovery period was divided into two 15-min increments, with the first 15 min including the therapy dog and the last 15 min without the dog. All sessions were video recorded.

For both study groups, the therapy dogs were accompanied by their handler at all times. The dog remained on a 6′ foot leash throughout the experiment. For the social + physical touch group, participants were allowed and actively encouraged to socialize and touch the dog at any point during the experiment. Participants in the social group were instructed to only talk to the dog and not to touch them. Handlers were also instructed to minimize engagement with the participants to avoid adding additional interactions that could possibly influence the results.

### 2.3. Measures

#### 2.3.1. Behavioral Observations

This current study used the behavioral data coded from Dowling-Guyer et al.’s (2024) adolescent and dog ethograms using video data from the experiment. Dowling-Guyer et al. (2024) coded behaviors in either counts (frequency) or durations (seconds). A total of 25 adolescent behaviors and 28 dog behaviors were extracted for initial review. We stratified the coded behavioral data further into groups by stress-linked or affiliative responses (Epstein et al., 2021; Lee et al., 2022). Behaviors that were not observed in both experimental groups (e.g., touching the dog) were removed from this analysis. This resulted in a total of nine adolescent behaviors for analysis, including: biting nails, general fidgeting, repositioning, touching face, yawning, talking to the dog handler and the dog, laughing, and smiling. The final eight dog behaviors were: panting, shake-off, shift within position, yawning, turning head towards participant, pawing at participant, sniffing participant, and wagging tail. As reported in Dowling-Guyer et al. (2024), interrater reliability for behavior coding was excellent (0.81 to 1.00 for human behaviors and 0.62 to 1.00 for dog behaviors).

#### 2.3.2. Self-Reported Anxiety

To capture momentary anxiety during the stress task, adolescents answered the short form version of the State-Trait Anxiety Inventory (STAI) (Marteau & Bekker, 1992), which is a widely used measure of acute anxiety (Seligman et al., 2004). The measure included 6 questions on momentary anxiety-related feeling states, which were adapted to a 3-point scale from 1 to 3 (e.g., I feel… very calm (1), calm (2), not calm (3)). Items were summed to create a score ranging from 6 to 18, with higher scores representing higher self-reported anxiety. This modification of the short form was done to ensure the feasibility of repeated measurements during the experiment, which resulted in Cronbach alpha scores ranging from 0.69 to 0.83 (see Mueller et al., 2021, for more details). STAI data were collected from participants via an iPad at six time points during the TSST-C (see Figure 1).

#### 2.3.3. Physiological Arousal

Physiological arousal in the form of continuous electrodermal activity (EDA) was captured using the Empatica E4, which is a wearable wristband sensor (Empatica Inc., n.d.). The wristbands were secured to the participant’s non-dominant hand. Participants were instructed to limit movement in this hand in order to reduce movement artifacts during data collection. Physiological data were collected throughout the experiment, from the beginning of the baseline phase to the end of the recovery period.

### 2.4. Data Analysis

Predictor and outcome data from both experimental (live therapy dog) conditions from Mueller et al. (2021) were extracted for analysis. As this study’s purpose was to examine the behaviors of live therapy dogs and adolescent participants, analyzing and pooling the data together from these two groups were necessary in order to test our hypotheses. As the two experimental conditions varied by their instructions to touch the therapy dog or not, the touch dog behavioral variable was removed from analysis. Upon removal of the touch dog variable, the data across experimental conditions became suitable to pool together into a single data set.

A priori power analyses were calculated with G*Power v3.1.9.7 (Faul et al., 2009) prior to coding dog and human behaviors, using a conservative effect size (*f*^2^) of 0.29, which was estimated from prior research, including Mueller et al. (2021). After specifying a linear multiple regression, *R*^2^ increase, fixed model with two tested (e.g., behaviors) and three total predictor variables (we expected to include condition as a predictor but after pooling the live dog conditions, this was not necessary), and with power = 0.80 and alpha = 0.05, we calculated a sample size of 37 would be needed to detect significant relationships between behaviors and stress measures. This analysis suggests that our sample size (*n* = 50) would be sufficient for our proposed analyses, depending on the number of behavioral predictors that emerged from the data. IBM SPSS Statistics for Windows, Version 30.0 II (IBM Corporation, n.d.) was used for analysis. EDA data were extracted from Empatica (Empatica Inc., n.d.), and both EDA and STAI data were cleaned and coded into peak and recovery scores. EDA was processed into segments of five minutes each that represented the average EDA during these segments (baseline, stressor, recovery). EDA peak scores were determined by subtracting the mean baseline segment from the mean stressor segment (i.e., stressor—baseline). EDA recovery scores were calculated by subtracting the mean EDA during the first recovery period from the mean EDA during the stressor (i.e., stressor—recovery) (see Figure 1). Similarly, the STAI peak scores were determined by subtracting baseline STAI (timepoint 1) from STAI at the end of the stressor (timepoint 4). STAI recovery was calculated by subtracting the first recovery period (timepoint 5) from STAI at the end of the stressor (timepoint 4) (see Figure 1). Descriptive statistics (mean, standard deviation, minimum, maximum) for EDA and STAI data were calculated.

The distributional properties of the final nine adolescent and eight dog behaviors were assessed prior to analysis. If the behaviors demonstrated a bimodal distribution, they were dichotomized, where 0 = behavior was absent, and 1 = behavior was present at some point during the experimental session. Behaviors coded in counts indicated the total number of instances in which the behavior occurred. Behaviors coded by duration reflected the length of time the behavior was recorded in seconds. Four of the final nine adolescent behaviors (biting nails, participant talks to handler, participant talks to dog, and laughing) and five of the eight dog behaviors (panting, shake-off, pawing at participant, sniffing participant, and wagging tail) were converted into binary variables. The remaining adolescent and dog behavioral items were kept as originally coded (count or duration). Frequencies of dichotomized behavioral data were calculated, as were descriptive statistics (mean, standard deviation, minimum, maximum) for the non-dichotomized behavioral data. Non-dichotomized behavioral data were standardized by converting to z scores for the regression models.

Linear regression modeling was used to test the predictive relationship between the standardized adolescent and dog stress-linked and affiliative behaviors as the predictor items to adolescent EDA and STAI peak and recovery outcomes. Assumption checks of linearity, homoscedasticity, and residual behaviors were assessed as appropriate for the model through visual inspection (i.e., scatterplots, histograms, p-p-plots), and independence of observations was confirmed through the Durbin–Watson statistic (Barker & Shaw, 2015). Outliers were also examined through scatterplots, and casewise diagnostics assessing individual behaviors falling outside of the range of ±3 standard deviations as the cutoff score. Few outliers emerged. However, given the nature of behavioral data, which often does not always fall within normal distribution ranges (Dowling-Guyer et al., 2024; Heit et al., 2024) and the study’s small sample size, the outliers were retained within the models.

Each of the standardized nine adolescent and eight dog behaviors was included in an individual linear model per outcome variable (peak EDA arousal, peak STAI, recovery EDA, and recovery STAI). Given the repeated measures within the small sample size, Cohen’s effects *f*^2^ was used to assess the local effect size of the individual predictors (Selya et al., 2012). This measure of effect size was used instead of a multiple comparison correction, as Cohen’s *f*^2^ provides a more detailed and sensitive representation of the magnitude of the effect within the relationship, compared to a multiple correction test that relies on a *p*-value cutoff score for determining significance.

## 3. Results

Frequencies and descriptive statistics for the binary and unstandardized behavioral predictors and outcome variables are presented in Table 1, Table 2 and Table 3.

Results from the linear regression models are presented in Table 4, Table 5, Table 6 and Table 7. The majority of the models did not find that the behavioral items significantly predicted EDA or STAI outcomes. However, two behaviors in the dog stress-linked behavioral models were significantly associated with EDA or STAI results. The first was a weak but significant negative association (B = −0.540) between standardized counts of the therapy dog yawning and EDA recovery (*F* [1, 48] = 4.221, *p* = 0.045), accounting for 8% of the model’s explained variability. This result signals that every additional count of yawning in dogs predicted a smaller decrease (i.e., less recovery) in the participant’s return to EDA baseline levels. This finding aligns with the anticipated results from our first hypothesis, suggesting that this stress-linked behavior may be associated with slower recovery after the stress task. However, this relationship between dog yawning and recovery to physiological baseline levels of EDA had a small to medium Cohen’s *f*^2^ effect size of 0.09 (Ellis, 2010) and thus should be interpreted with caution.

A second significant finding emerged opposite to the expected direction identified in our first hypothesis. We found a significant negative relationship (B = −1.550) between the dichotomized dog shake-off behavior and self-reported peak anxiety levels (*F* [1, 45] = 6.716, *p* = 0.013, r^2^ = 0.130) with a moderate effect size (*f*^2^ = 0.15). This indicates that in sessions where the dog exhibited the shake-off behaviors, adolescents reported lower levels of self-reported peak anxiety as compared to sessions where the dog did not shake off.

## 4. Discussion

AAI is gaining traction as a potential treatment option for adolescent social anxiety, with prior research linking interactions with dogs to stress buffering effects in the form of social support (Brandão et al., 2025; Kertes et al., 2017). However, there is a notable gap in the literature on the behavioral drivers that may result in anxiety reduction for adolescents participating in AAIs. The primary goal of this study was to investigate the relationship between adolescent and dog behaviors and changes in adolescent physiological arousal and self-reported anxiety during an experimental AAI. By examining the behaviors of adolescents and dogs, we anticipated that we would gain insight into behavioral dynamics that could be related to adolescent treatment effectiveness, as well as well-being outcomes for both species. Specifically, we expected that adolescent and dog stress-linked behaviors would be related to greater levels of adolescent peak reactivity to a stress task and lower levels of recovery after the stressor. In contrast, we hypothesized that adolescent and dog affiliative behaviors would be linked to lower levels of peak reactivity and greater return to baseline self-reported anxiety and physiological arousal levels. Overall, our results generally did not support our hypotheses. There was no evidence of a significant relationship between affiliative behaviors displayed by either the adolescent or the dog to adolescent arousal or self-reported anxiety. Surprisingly, our results did not indicate that adolescent stress behaviors were linked to self-reported anxiety or physiological arousal. However, a few significant relationships emerged between dog stress-linked behaviors and measures of adolescent physiological arousal and self-reported anxiety.

Our first finding was a significant but small in magnitude association in the anticipated direction between the therapy dog yawning and the adolescent’s physiological arousal recovery. In simple terms, more yawning in the therapy dogs predicted smaller returns to baseline physical arousal levels in the adolescent (less recovery). This finding may reflect the role that yawning behaviors have on the dog’s ability to cope with stress. This form of coping behavior, which is commonly referred to as a displacement behavior, has been connected to a dog’s management of stress within social settings (Kuhne et al., 2012; Pedretti et al., 2023). Dog yawning as a displacement behavior has been noted to appear across a variety of settings, including within interactions between conspecifics (Pedretti et al., 2023) and humans (Buttner & Strasser, 2014), as well as being observed in response to an environmental stressor (Beerda et al., 2000). However, the role that the yawning behavior has as a coping response for dogs within AAIs is still inconclusive. A recent study by Erichsmeier et al. (2025) found that therapy dog displacement behaviors, which included yawning, were minimally observed with AAIs. However, Townsend and Gee (2021) noted in their case study on dog welfare outcomes when involved in AAIs that yawning behaviors were paired with other stress-linked responses, like avoidance of the client. Given this, it is possible that the therapy dogs in this present study exhibited yawning behaviors as an attempt to cope with the adolescent’s lower ability to self-regulate (i.e., return to baseline physiological arousal levels). However, because there has not yet been a clear link establishing yawning behaviors to therapy dog stress within AAIs, it is possible that the reason the dogs who yawned in this current study was due to an alternative explanation other than them displacing stress.

Another possible explanation for this first finding is related to the theory of emotional contagion within human–dog relationships. This theory posits that both human and nonhuman animals have an automatic mimicry of emotional responses within or between species interactions that relates to an innate self-preservation response (Pérez-Manrique & Gomila, 2022). Emotional contagion is considered one of the most primitive forms of empathy (Pérez-Manrique & Gomila, 2022), and has been identified as a prominent theory of the mechanistic drivers behind human–animal interactions (Barcelos et al., 2023). Indeed, there is a growing literature base supporting that emotional mimicry has a role within human–dog stress-related behaviors (Duranton & Gaunet, 2015; Pérez-Manrique & Gomila, 2022; Rial et al., 2025; Sundman et al., 2019). For example, Rial et al. (2025) found in their trial on dogs’ emotional contagion responses to their caregivers that when a dog’s caregiver was a victim of a conflict, their dogs showed more instances of affiliative behaviors with the caregiver, including longer eye contact, more physical contact, and remaining in close proximity, compared to those conditions in which their caregiver behaved as the conflict aggressor. Thus, there is a theoretical basis to hypothesize that in this present study, the positive relationship between adolescent and therapy dog yawning behaviors could be linked to an emotional contagion response.

The role of emotional contagion in AAI is an important yet complicated one to unpack when considering dog welfare outcomes. As the field of HAI research continues to investigate AAI best practices, it is crucial to understand which types of therapeutic interactions prompt dog stress, and ideally, which are linked to positive welfare states (Mellor, 2016; Mellor et al., 2020). If a pattern can be identified in the types of interactions that are associated with stress-linked responses in therapy dogs, those findings can be used to inform best practices for AAI sessions to avoid those stress-inducing dynamics for the dog. Emotional contagion theory adds a layer of complexity in understanding a therapy dog’s experience of stress in AAIs. For example, when observing a dog’s stress response, is that reaction related to the dog’s individual experience of stress (i.e., displacement of stress), or is it because they are reflecting the client’s stress (i.e., emotional contagion)? Buttner and Strasser (2014) found that shelter dog yawning responses were linked to elevated levels of cortisol after being exposed to a human yawn. Thus, it is possible that these two responses, that of displacement behaviors of stress and emotional contagion of stress, are intertwined within the context of human–dog interactions. In order to support best practice development in AAIs that result in mutually beneficial outcomes for both the human and dog participants, continued research is needed to investigate the concepts of displacement behaviors and emotional contagion, and how they emerge within AAIs.

Our second significant finding revealed a negative association between therapy dog shaking off behavior and adolescent self-reports of peak anxiety during the social stress task. In other words, shake-off behavior in the therapy dogs predicted lower levels of self-reported peak anxiety in adolescents compared to when the shake-off behaviors did not occur. This finding was in the opposite direction of our hypothesis, where we anticipated that we would see higher levels of adolescent peak reactivity when dogs were demonstrating stress-linked behaviors. This result does align somewhat with previous research by Glenk et al. (2014), who found that shake-off behaviors were correlated with lower cortisol levels in the therapy dogs who were participating in an AAI. Glenk et al. (2014) suggest that shake-off behavior was not necessarily a direct response to stress but instead may have been a transition behavior in relation to a potentially stressful situation. In fact, the notion of a shake-off behavior as an interpersonal transitional response is supported by a recent study from Bryce et al. (2024), who investigated the role that shake-off behaviors have amongst dogs within a natural social setting. They concluded that dogs’ shake-off behaviors were not stress-linked per se, but instead a signal of transition to a new behavior, though the nature of the post shake-off response was not examined. These results suggest that shake-off behaviors could be a marker of a transition to a new behavior during adolescent–dog interactions in AAI and not necessarily a reflection of the type of behaviors or emotional state that warrant the shake-off. Given the role of shake-off behaviors as a transitional response, it would be useful for future research to examine the timeline before and after the shake-off behaviors to understand the temporal dynamics that may have prompted that response.

### Limitations and Future Research

There are several limitations that could be contributing to the null and weak findings. First is the small sample size within this study, of both participants and the frequency of certain behaviors. While our a priori power analysis, which assumed three predictors, indicated that 50 participants would be sufficient to detect effects, due to the exploratory nature of the study, we identified more than three predictors to include in the models. Furthermore, our behaviors were clustered within four dogs. Due to the small number of dogs as well as the low frequency of many behaviors, we did not have the power to conduct multilevel modeling to account for the nested nature of the data, which is a limitation of this exploratory study. Therefore, the analyses overall may have been underpowered to detect relationships between behaviors and outcomes. Because this study used an existing dataset from a prior experiment, we did not have control over the number of dogs in the analysis. Future research should explore the between-dog differences in behavioral patterns and frequencies.

The lack of variability within behaviors could have restricted an accurate representation of the relationship between adolescent and dog behaviors and adolescent reactivity measures. As instances of individual behavioral observations were aggregated across all time points within the study. This adds an additional limitation as the behavioral scores do not indicate when the observation happened during the study, but instead acted as a signal that the behavior occurred at some point during the TSST-C. A combination of infrequent behaviors and the inability to identify the behavioral sequence of events limits this study’s ability to draw any causal inferences. Future research should consider replicating with a larger, representative sample, as well as examining the temporal dynamics within the adolescent–dog interactions that lead to stress-linked or affiliative responses, and how those responses predict anxiety reduction outcomes.

While the sample size and aggregation of data across the study limit causal inferences, the inclusion of trained therapy dogs could also be another limiting factor in the study’s results. As mentioned in Dowling-Guyer et al. (2024), the dogs who were included in this study have undergone intensive training in order to become certified to engage in therapeutic interactions. Part of the process in becoming registered as a therapy team is that the dog displays minimal reactivity when engaging in a variety of settings, which can include scenarios in which they encounter a person who is experiencing anxiety (Pet Partners, 2018). Additionally, the dogs in this study had prior exposure to working in therapeutic settings; thus, it is possible that there could be a degree of desensitization that is limiting their stress response.

The therapy dogs also remained on leash for the duration of the experiment, and this restrictive gear could have had a role in shaping the results, as these restraints can often be a signal to the dog that they are engaging in a work-related activity. It is possible that once the associated “on-duty” gear is removed, their behavior could have changed. In summary, this study highlights a challenge within research on therapy dog behaviors, as it would not be ethical to pursue attempts to increase therapy dog stress for the sake of experimentation or to replicate studies with untrained therapy dogs. However, it would be insightful to consider a study design that examines the behavioral responses of adolescent–dog interactions with both familiar and unfamiliar therapy dogs. As stress-linked and affiliative responses from dogs may also vary based on how familiar they are with the person they are interacting with (Kuhne et al., 2014), this would be a worthy endeavor to examine the role that familiarity has on both human and dog outcomes within AAI, as long as measures are taken to ensure the dog’s welfare. Furthermore, our study did not examine whether adolescent awareness and interpretation of dog stress behaviors had an impact on our findings. As prior research has identified that subtle behavioral signs of dog stress are often missed (Mariti et al., 2012; Mariti et al., 2015), future investigations should consider the effect that awareness and interpretation of dog stress behaviors have on both dog and adolescent participant outcomes within the context of AAIs.

The effect of the therapy dog handler was not investigated in this study, which is another limitation. The therapy dog handler plays a crucial role in monitoring and ensuring the dog’s welfare throughout the interaction (Glenk, 2017; Mignot et al., 2022). Because they are involved in the therapeutic interaction, it is plausible that they can have an influence on the outcomes. Indeed, research in this area is growing in attention, with a recent study by Nilsson et al. (2024) reporting that the handler’s physical and tactical engagement with the participant had a significant role in shaping the client outcomes from the AAIs. Furthermore, as Sidel et al. (2025) stated in their comparable trial on AAIs and dog welfare outcomes, it is possible that dog stress responses while in an AAI could be attributed to their past experiences, or even to the environmental conditions themselves. Also related to potential environmental influences, it is possible that internal (caffeine consumption, hydration) or external (room temperature) factors could have increased variability in the EDA data from participant to participant, which could obscure our ability to detect relationships between EDA and behaviors. Therefore, future research should consider all aspects of the therapeutic interaction while studying AAIs within an experimental setting. From the environmental conditions in which the intervention occurs, the therapy dog’s history, as well as the handler’s influence on both the dog and the client outcomes, future trials should attempt to control for all possible effects when studying AAIs in an experimental setting.

There are also limitations to using behavioral data. While the behavior coding demonstrated strong interrater reliability (Dowling-Guyer et al., 2024), the behavioral data captured were limited to the video camera’s angle and frame. It is possible that additional behaviors occurred outside of the video camera’s recording window. Additionally, as some of the behavioral variables were dichotomized, another potential limitation is that some of the variations and nuances across these variables could have been lost when converted into binary structures.

Furthermore, therapy dog outcome data were limited to behavioral observation through video recording. It is possible that using additional measures to capture dog outcomes, such as physiological stress markers, might have given us more nuanced insights into the dog’s experiences during the session. There are a variety of biological indicators that can be used to examine human and dog stress reactivity in therapeutic settings, and each have their own benefits or limitations (Gandenberger et al., 2022). Thus, future research should examine the most appropriate physiological stress arousal biomarkers to incorporate into ongoing research on the human–dog behavioral drivers of anxiety reduction outcomes in AAIs.

It is also possible that grouping behaviors by stress-linked or affiliative is not a sufficient categorization structure, as a single behavior could have multiple meanings (as previously discussed with regard to the shake-off behavior). Especially in consideration of dog behaviors, researchers are still grappling with categorizing the meaning of ambiguous behaviors. Furthermore, while grouping the behavioral data as stress-linked or affiliative was generated based on previous research, the data collected for the present study were not validated to ensure that they fit with the categorization structure. Additional research on the role of stress-linked and affiliative behaviors across human–dog interactions within AAIs continues to be an area of needed investigation. It is recommended for future research in this area to consider adding methods of validating the classification of behaviors as stress-linked and affiliative responses to examine the fidelity and feasibility in capturing both human and dog outcomes within AAIs.

Finally, we anticipate that both our demonstrated significant effects and null results could be of use to both researchers and practitioners in the field of AAIs. To our knowledge, our study is one of the first that examines both stress-linked and affiliative behaviors between adolescents and therapy dogs within an experimental AAI to address social anxiety. As future replication of this design is encouraged with larger, more diverse samples, our procedures and methods related to testing both adolescent and dog outcomes related to stress-linked and affiliative behaviors could aid in future replication on this topic. Furthermore, due to limited insight into dog behavioral dynamics within AAIs, and more specifically, how those relate to adolescent changes in stress reactivity, this research could be of use to practitioners and researchers working to define best practices in AAI for adolescents with social anxiety.

## 5. Conclusions

Overall, this study’s findings mostly did not support the hypothesis regarding relationships between adolescent and dog affiliative behaviors and adolescent anxiety outcomes. Additionally, our results did not support the hypothesis that stress-linked behaviors in participants and therapy dogs are linked to higher levels of self-reported anxiety and physiological arousal or lower abilities to recover from peak reactivity. Thus, no conclusions can be drawn regarding the role that adolescent or therapy dog stress-linked or affiliative behaviors have on the treatment effectiveness of AAI for adolescents with social anxiety.

Our results did signal two significant associations between dog stress behaviors and adolescent physiological arousal and self-reported anxiety. The first demonstrated a small and relatively weak significant relationship between yawning in dogs and lower levels of returning to baseline physiological arousal levels in adolescents. The second significant finding indicated an inverse relationship between shake-off behaviors in dogs and lower levels of peak self-reported anxiety in adolescents. However, given the limitations of a small size, minimal stress-linked behaviors in the dogs, and overall limitations of behavioral data, no conclusive inferences can be drawn. It is recommended for future research to replicate the study design with a larger sample, examine the temporal dynamics within each condition, and to investigate the role that familiar compared to unfamiliar dogs can have on treatment outcomes in AAI. Additionally, it would be fruitful for future studies to incorporate different methods of collecting stress-linked and affiliative responses using psychophysiological data for both the adolescent and the dog involved in the AAI.

## Figures and Tables

**Table 1:** Frequencies of binary stress-linked and affiliative behaviors within adolescent–dog dyads (*N* = 50).

Individual Behaviors		*n*	%
Adolescent Stress Behaviors	Bites Nails (Duration)	27	54.0%
Adolescent Affiliative Behaviors	Participant Talks to Handler (Duration)	47	94.0%
	Laughing (Count)	45	90.0%
	Participant Talks to Dog (Duration)	32	64.0%
Dog Stress-Linked Behaviors	Panting (Duration)	7	14.0%
	Shake Off (Duration)	22	44.0%
Dog Affiliative Behaviors	Pawing at Participant (Count)	12	24.0%
	Sniff Participant (Duration)	35	70.0%
	Wagging tail (Duration)	26	52.0%

Note. Behaviors were originally coded in durations or counts by Dowling-Guyer et al. (2024). Frequencies of each behavior are based on individual adolescent/dog pairings (*N* = 50), not based on the number of dogs in the sample.

**Table 2:** Descriptive statistics of duration and count stress-linked and affiliative behaviors within adolescent–dog dyads (*N* = 50).

Individual Behaviors		Mean	SD	Min	Max
Adolescent Stress Behaviors	General Fidget (Duration, Seconds)	824.44	493.84	0.00	2555.44
	Reposition (Count)	96.54	49.07	0.00	214.00
	Touch Face (Count)	29.22	20.93	0.00	93.00
	Yawn (Count)	3.44	3.66	0.00	16.00
Adolescent Affiliative Behaviors	Smiling (Count)	25.84	14.33	0.00	69.00
Dog Stress-Linked Behaviors	Shift Within Position (Count)	54.18	39.36	1.00	212.00
	Yawning (Count)	1.92	2.35	0.00	11.00
Dog Affiliative Behaviors	Turn Head Towards Participant (Duration, Seconds)	37.21	41.16	0.00	210.10

Note. Descriptive statistics are based on the number of times or the number of seconds each behavior occurred in individual adolescent/dog pairings (*N* = 50), not based on the number of dogs in the sample. Descriptive statistics are of unstandardized variables.

**Table 3:** Descriptive statistics for adolescent electrodermal activity (EDA) and State-Trait Anxiety Inventory (STAI) measures.

Stress Reactivity	*n*	Mean	SD	Min	Max
EDA Peak	50	3.23	2.36	−0.92	8.61
EDA Recovery	50	1.35	1.89	−4.29	6.93
STAI Peak Anxiety	47	5.34	2.15	2.00	11.00
STAI Anxiety Recovery	49	4.39	2.29	−1.00	12.00

Note. EDA data are measured in microseimens. EDA peak = average EDA during the stressor period minus average EDA during the baseline period. EDA recovery = average EDA during recovery with dog minus average EDA during stressor. STAI peak = STAI at end of stressor (timepoint 4) minus STAI baseline (timepoint 1). STAI recovery = STAI at end of stressor (timepoint 4) minus the STAI score at end of recovery period with dog (timepoint 5) (See Figure 1).

**Table 4:** Adolescent stress behavioral predictors of adolescent stress reactivity: Linear regression models.

Outcome	Adolescent Stress Behaviors	Estimate (B)	*SE*	*β*	*R* ^2^	*f* ^2^	95% CI		*p*
							*LL*	*UL*	
EDA Peak	Bites Nails (Duration, Binary)	−0.327	0.674	−0.070	0.005	0.00	−1.682	1.028	0.630
	General Fidget (Duration, Continuous)	−0.055	0.340	−0.024	0.001	0.00	−0.739	0.628	0.871
	Reposition (Count, Continuous)	−0.270	0.338	−0.115	0.013	0.01	−0.949	0.410	0.428
	Touch Face (Count, Continuous)	−0.341	0.337	−0.145	0.021	0.02	−1.018	0.336	0.316
	Yawn (Count, Continuous)	0.148	0.340	0.063	0.004	0.00	−0.535	0.830	0.666
EDA Recovery	Bites Nails (Duration, Binary)	−0.231	0.543	−0.061	0.004	0.00	−1.323	0.861	0.673
	General Fidget (Duration, Continuous)	0.022	0.274	0.011	0.000	0.00	−0.529	0.572	0.938
	Reposition (Count, Continuous)	0.508	0.264	0.267	0.072	0.08	−0.023	1.038	0.060
	Touch Face (Count, Continuous)	0.329	0.270	0.173	0.030	0.03	−0.213	0.872	0.229
	Yawn (Count, Continuous)	0.182	0.273	0.096	0.009	0.01	−0.367	0.730	0.009
STAI Peak	Bites Nails (Duration, Binary)	0.127	0.635	0.030	0.001	0.00	−1.152	1.406	0.842
	General Fidget (Duration, Continuous)	−0.315	0.311	−0.149	0.022	0.02	−0.942	0.312	0.317
	Reposition (Count, Continuous)	−0.183	0.318	−0.085	0.007	0.01	−0.823	0.458	0.568
	Touch Face (Count, Continuous)	0.080	0.319	0.037	0.001	0.00	−0.562	0.723	0.802
	Yawn (Count, Continuous)	0.533	0.318	0.243	0.059	0.06	−0.107	1.173	0.101
STAI Recovery	Bites Nails (Duration, Binary)	0.485	0.659	0.107	0.011	0.01	−0.840	1.810	0.465
	General Fidget (Duration, Continuous)	−0.319	0.327	−0.141	0.020	0.02	−0.978	0.339	0.335
	Reposition (Count, Continuous)	−0.186	0.336	−0.081	0.006	0.01	−0.861	0.489	0.582
	Touch Face (Count, Continuous)	−0.046	0.333	−0.020	0.000	0.00	−0.716	0.624	0.891
	Yawn (Count, Continuous)	0.371	0.329	0.162	0.026	0.03	−0.291	1.033	0.266

Note. Outcome variables = Electrodermal activity (EDA); State-Trait Anxiety Inventory (STAI). Peak = highest point of anxiety during experiment, recovery = return from peak anxiety to baseline 15 min post-experiment. B = unstandardized beta. β = standardized beta. *LL* = lower limit; *UL* = upper limit.

**Table 5:** Adolescent affiliative behavioral predictors of adolescent stress reactivity: Linear regression models.

Outcome	Adolescent Affiliative Behaviors	Estimate (B)	*SE*	*β*	*R* ^2^	*f* ^2^	95% CI		*p*
							*LL*	*UL*	
EDA Peak	Participant Talks to Handler (Duration, Binary)	0.257	1.418	0.026	0.001	0.00	−2.593	3.108	0.857
	Laughing (Count, Binary)	−0.090	1.123	−0.012	0.000	0.00	−2.347	2.167	0.937
	Smiling (Count, Continuous)	−0.132	0.340	−0.056	0.003	0.00	−0.815	0.551	0.699
	Participant Talks to Dog (Duration, Binary)	0.075	0.702	0.016	0.000	0.00	−1.335	1.486	0.915
EDA Recovery	Participant Talks to Handler (Duration, Binary)	0.767	1.137	0.097	0.009	0.01	−1.518	3.052	0.503
	Laughing (Count, Binary)	0.160	0.904	0.026	0.001	0.00	−1.657	1.977	0.860
	Smiling (Count, Continuous)	0.320	0.270	0.169	0.029	0.03	−0.222	0.863	0.241
	Participant Talks to Dog (Duration, Binary)	0.737	0.555	0.188	0.035	0.04	−0.379	1.853	0.191
STAI Peak	Participant Talks to Handler (Duration, Binary)	0.356	1.570	0.034	0.001	0.00	−2.806	3.517	0.822
	Laughing (Count, Binary)	0.605	1.024	0.088	0.008	0.01	−1.458	2.668	0.558
	Smiling (Count, Continuous)	0.095	0.313	0.045	0.002	0.00	−0.535	0.725	0.763
	Participant Talks to Dog (Duration, Binary)	0.349	0.658	0.079	0.006	0.01	−0.976	1.674	0.598
STAI Recovery	Participant Talks to Handler (Duration, Binary)	0.404	1.670	0.035	0.001	0.00	−2.954	3.763	0.810
	Laughing (Count, Binary)	0.209	1.092	0.028	0.001	0.00	−1.987	2.405	0.849
	Smiling (Count, Continuous)	0.126	0.331	0.056	0.003	0.00	−0.539	0.792	0.704
	Participant Talks to Dog (Duration, Binary)	−0.090	0.686	−0.019	0.000	0.00	−1.469	1.290	0.897

Note. Outcome variables = Electrodermal activity (EDA); State-Trait Anxiety Inventory (STAI). Peak = highest point of anxiety during experiment, recovery = return from peak anxiety to baseline 15 min post-experiment. B = unstandardized beta. β = standardized beta. *LL* = lower limit; *UL* = upper limit.

**Table 6:** Dog stress-linked behavioral predictors of adolescent stress reactivity: Linear regression models.

Outcome	Dog Stress-Linked Behaviors	Estimate (B)	*SE*	*β*	*R* ^2^	*f* ^2^	95% CI		*p*
							*LL*	*UL*	
EDA Peak	Panting (Duration, Binary)	0.591	0.967	0.088	0.008	0.01	−1.353	2.535	0.544
	Shake Off (Duration, Binary)	−1.089	0.660	−0.232	0.054	0.06	−2.416	0.238	0.105
	Shift Within Position (Count, Continuous)	−0.132	0.340	−0.056	0.003	0.00	−0.815	0.550	0.698
	Yawning (Count, Continuous)	−0.023	0.340	−0.010	0.000	0.00	−0.707	0.661	0.947
EDA Recovery	Panting (Duration, Binary)	−0.738	0.774	−0.136	0.019	0.02	−2.294	0.819	0.346
	Shake Off (Duration, Binary)	−0.214	0.545	−0.056	0.003	0.00	−1.311	0.883	0.697
	Shift Within Position (Count, Continuous)	−0.449	0.266	−0.236	0.056	0.06	−0.984	0.086	0.098
	**Yawning (Count, Continuous)**	**−0.540**	**0.263**	**−0.284**	**0.081**	**0.09**	**−1.068**	**−0.012**	**0.045**
STAI Peak	Panting (Duration, Binary)	−0.199	0.950	−0.031	0.001	0.00	−2.112	1.713	0.835
	**Shake Off (Duration, Binary)**	**−1.550**	**0.598**	**−0.360**	**0.130**	**0.15**	**−2.755**	**−0.345**	**0.013**
	Shift Within Position (Count, Continuous)	0.025	0.312	0.012	0.000	0.00	−0.604	0.654	0.937
	Yawning (Count, Continuous)	−0.030	0.316	−0.014	0.000	0.00	−0.666	0.606	0.925
STAI Recovery	Panting (Duration, Binary)	0.508	1.006	0.073	0.005	0.01	−1.515	2.531	0.616
	Shake Off (Duration, Binary)	0.071	0.668	0.016	0.000	0.00	−1.272	1.415	0.915
	Shift Within Position (Count, Continuous)	0.398	0.326	0.176	0.031	0.03	−0.257	1.053	0.228
	Yawning (Count, Continuous)	0.139	0.332	0.061	0.004	0.00	−0.529	0.808	0.667

Note. Outcome variables = Electrodermal activity (EDA); State-Trait Anxiety Inventory (STAI). Peak = highest point of anxiety during experiment, recovery = return from peak anxiety to baseline 15 min post-experiment. B = unstandardized beta. β = standardized beta. *LL* = lower limit; *UL* = upper limit. Bolded rows = *p* < 0.05.

**Table 7:** Dog affiliative behavioral predictors of adolescent stress reactivity: Linear regression models.

Outcome	Dog Affiliative Behaviors	Estimate (B)	*SE*	*β*	*R* ^2^	*f* ^2^	95% CI		*p*
							*LL*	*UL*	
EDA Peak	Turn Head Towards Participant (Duration, Continuous)	−0.356	0.336	−0.151	0.023	0.02	−1.032	0.321	0.296
	Pawing at Participant (Count, Binary)	0.618	0.784	0.113	0.013	0.01	−0.958	2.193	0.434
	Sniffing Participant (Duration, Binary)	0.205	0.734	0.040	0.002	0.00	−1.271	1.682	0.781
	Wagging Tail (Duration, Binary)	−0.215	0.673	−0.046	0.002	0.00	−1.569	1.139	0.751
EDA Recovery	Turn Head Towards Participant (Duration, Continuous)	−0.041	0.274	−0.022	0.000	0.00	−0.592	0.509	0.880
	Pawing at Participant (Count, Binary)	−0.162	0.635	−0.037	0.001	0.00	−1.438	1.114	0.800
	Sniffing Participant (Duration, Binary)	−0.462	0.588	−0.113	0.013	0.01	−1.645	0.720	0.436
	Wagging Tail (Duration, Binary)	−0.627	0.535	−0.167	0.028	0.03	−1.703	0.449	0.247
STAI Peak	Turn Head Towards Participant (Duration, Continuous)	−0.233	0.315	−0.110	0.012	0.01	−0.867	0.401	0.436
	Pawing at Participant (Count, Binary)	−0.682	0.742	−0.136	0.018	0.02	−2.176	0.812	0.363
	Sniffing Participant (Duration, Binary)	−0.185	0.680	−0.041	0.002	0.00	−1.554	1.183	0.786
	Wagging Tail (Duration, Binary)	−0.471	0.631	−0.110	0.012	0.01	−1.743	0.801	0.460
STAI Recovery	Turn Head Towards Participant (Duration, Continuous)	0.071	0.332	0.031	0.001	0.00	−0.596	0.739	0.830
	Pawing at Participant (Count, Binary)	0.555	0.788	0.102	0.010	0.01	−1.030	2.140	0.485
	Sniffing Participant (Duration, Binary)	−0.594	0.712	−0.121	0.015	0.02	−2.060	0.838	0.408
	Wagging Tail (Duration, Binary)	−0.908	0.649	−0.200	0.040	0.04	−2.214	0.398	0.168

Note. Outcome variables = Electrodermal activity (EDA); State-Trait Anxiety Inventory (STAI). Peak = highest point of anxiety during experiment, recovery = return from peak anxiety to baseline 15 min post-experiment. B = unstandardized beta. β = standardized beta. *LL* = lower limit; *UL* = upper limit.

## Data Availability

The data from the original experimental study are openly available in the Open Science Framework at https://osf.io/w7k8p/?view_only=965f3d7390d7434895216fe8b88a2160 (accessed on 15 January 2026). Video data files are not publicly available due to the identifying nature of the videos (i.e., participant faces). Video code data are available upon request from the corresponding author.

## Abbreviations

The following abbreviations are used in this manuscript:

AAIs	Animal-Assisted Interventions
HAI	Human–Animal Interactions
EDA	Electrodermal Activity
STAI	State-Trait Anxiety Inventory
